# Mild hypothermia improves neurological outcome in mice after cardiopulmonary resuscitation through Silent Information Regulator 1-actviated autophagy

**DOI:** 10.1038/s41420-019-0209-z

**Published:** 2019-08-13

**Authors:** Hongyan Wei, Meixian Yin, Yuanzheng Lu, Yan Yang, Bo Li, Xiao-Xing Liao, Gang Dai, Xiaoli Jing, Yan Xiong, Chunlin Hu

**Affiliations:** 1 Department of Emergency, the First Affiliated Hospital, Sun Yat-sen University, Guangzhou, 510080 China; 2 NHC Key Laboratory of Assisted Circulation, Sun Yat-sen University, Guangzhou, 510080 China; 3 Department of Emergency, the Seventh Affiliated Hospital, Sun Yat-sen University, Shenzhen, 518107 China

**Keywords:** Cell death in the nervous system, White matter injury

## Abstract

Mild hypothermia treatment (MHT) improves the neurological function of cardiac arrest (CA) patients, but the exact mechanisms of recovery remain unclear. Herein, we generated a CA and cardiopulmonary resuscitation (CPR) mouse model to elucidate such function. Naïve mice were randomly divided into two groups, a normothemia (NT) group, in which animals had normal body temperature, and a MHT group, in which animals had a body temperature of 33 °C (range: 32–34 °C), after the return of spontaneous circulation (ROSC), followed by CA/CPR. MHT significantly improved the survival rate of CA/CPR mice compared with NT. Mechanistically, MHT increased the expression of Silent Information Regulator 1 (Sirt1) and decreased P53 phosphorylation (p-P53) in the cortex of CA/CPR mice, which coincided with the elevated autophagic flux. However, Sirt1 deletion compromised the neuroprotection offered by MHT, indicating that Sirt1 plays an important role. Consistent with the observations obtained from in vivo work, our in vitro study utilizing cultured neurons subjected to oxygen/glucose deprivation and reperfusion (OGD/R) also indicated that Sirt1 knockdown increased OGD/R-induced neuron necrosis and apoptosis, which was accompanied by decreased autophagic flux and increased p-P53. However, the depletion of P53 did not suppress neuron death, suggesting that P53 was not critically involved in MHT-induced neuroprotection. In contrast, the application of autophagic inhibitor 3-methyladenine attenuated MHT-improved neuron survival after OGD/R, further demonstrating that increased autophagic flux significantly contributes to MHT-linked neuroprotection of CA/CRP mice. Our findings indicate that MHT improves neurological outcome of mice after CA/CPR through Sirt1-mediated activation of autophagic flux.

## Introduction

Development of cardiopulmonary resuscitation (CPR) technology has significantly improved the success rate of the return of spontaneous circulation (ROSC) in patients with sudden cardiac arrest (CA)^1^. Still, <10% of such patients are discharged from the hospital; most patients in fact die from hypoxic ischemic brain damage after CPR^2^. Hence, suppressing the ischemia/reperfusion-induced brain injury is critical for reducing CPR-linked brain damage and subsequent death.

Recently, mild hypothermia treatment (MHT) has been shown to be effective in improving outcomes of patients after CA/CPR^3,4^. Mechanistically, MHT reduces the production of reactive oxygen species (ROS)^5^, ameliorates cellular dysfunction and apoptosis^6^, and reduces the metabolic rate in the brain, all of which collectively diminish oxygen consumption^7^. Another mechanism involving MHT-mediated neuroprotection is autophagy. Basal autophagy is a physiological process that removes bulk cellular components and organelles. However, autophagic flux may be altered, either increased or decreased, in response to external stimuli. A certain level of autophagy induced by mild or moderate cerebral ischemia or hypoxia offers protection against brain injury, decreases aging and increases tolerance to ischemia and hypoxia^8^, but excessive autophagy caused by hypoxia can lead to cell death^9^. Our previous study showed that activation of autophagy induces neurological protection in a rat CA model^10^. Also, both in vivo^11^ and in vitro^12^ studies have shown that increased autophagic flux is an important mechanism for MHT to reduce neuronal injury. However, how MHT regulates autophagy is still unclear.

Silent Information Regulator 1 (Sirt1) is abundant in the nervous system and has important neuroprotective functions. For instance, Sirt1 over-expression reduces neurodegeneration by suppressing neuronal apoptosis or deacetylating forkhead transcription factor 1 (FoxO1) and P53^13^. Sirt1 is also an important regulator of autophagy, as evidenced by the finding that Sirt1 regulates the autophagy-lysosome pathway by deacetylation of autophagy-related genes Atg5, Atg7, Atg8, and FoxO^14,15^. Consistent with the above findings, resveratrol, a Sirt1 activator, has been shown to induce autophagy in chronic myelogenous leukemia cells through JNK-mediated p62 expression and AMPK activation^16^. Recently, Sirt1 has been identified as a mediator of cerebral ischemia, with potential of being a therapeutic target^17^. Given the above findings, we hypothesized that Sirt1 may play an important role in MHT-regulated neuronal autophagy after CA/CRP. We examined our hypothesis in a mouse CA model and in the cultured neurons subjected to oxygenation deprivation and reoxygenation (OGD/R).

## Results

### MHT improved the survival rate and reduced apoptosis in the cortex of mice after CA/CPR

**Fig. 1:**
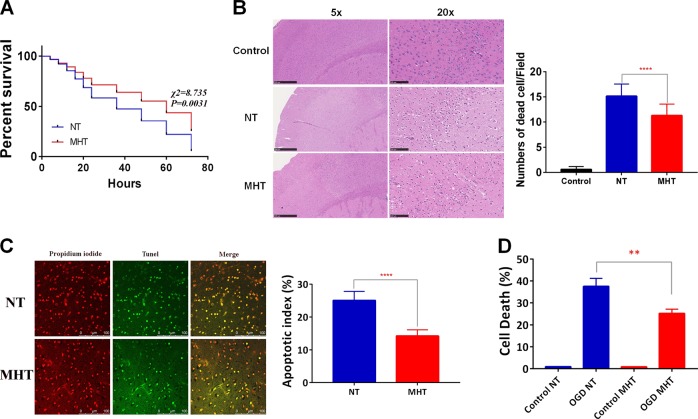
MHT rescues neurons from CA/CPR and OGD/R both in vivo and in vitro. **a** MHT improves the 72 h survival rate of mice after CA/CPR following ROSC from KCL-induced CA. **b** MHT reduces cell death in cortex after ROSC for 72 h. **c** MHT reduces apoptosis in cortex after ROSC for 72 h (scale bar 100 µm). **d** MHT reduces primary cortical neuron death after OGD/R for 24 h. NT normothermia, MHT mild hypothermia therapy, ROSC return of spontaneous circulation, OGD/R oxygen glucose deprivation and reperfusion. *p < 0.05; **p < 0.01; ***p < 0.001; ****p < 0.0001, vs. NT

Injection of KCL is an efficient way to induce CA and can also obtain a high ROSC rate in mice^18^. We established the animal CA model based on the protocol shown in the flow chart in Supplementary Fig. 1. All animals (18 in each group) achieved ROSC, but within 24 h after ROSC, approximately 40% of mice in the NT group and 30% mice in the MHT died; only two in the NT group and five in the MHT group survived past 72 h. The survival analysis demonstrated that MHT significantly improved the survival rate of mice (Fig. 1a). The CA and subsequent CPR induced severe injury to the neurons in the cortex of mice, as suggested by the observation that the number of dead cells in the MHT group was significantly lower than that in the NT group (Fig. 1b).

Epinephrine has been shown to significantly impair myocardial function by decreasing contractility and diastolic function. Hearts treated with epinephrine require a larger number of electrical countershocks to attain rhythm conversion due to its β receptor activation effects, by which the myocardial oxygen requirement is generall increased^19^. However, based on the dose used in this study, we did not see significant differences between the NT and MHT groups with regard to the basal physiological parameters (Supplementary Fig. 2). Neuronal apoptosis is one of the important manifestations induced by CA/CPR. Indeed, MHT significantly reduced apoptosis after ROSC compared with NT (14% vs. 25%) (Fig. 1c).

We further isolated primary cortex neurons from the P0-P1 newborn rats and seeded these cells onto six well plates at a concentration of 4–5 × 10^6^/well. Cytarabine (2 µmol) was used to inhibit glial cells after 6 h of planting. The neuronal specific markers Neun and map2 were used to identify the neurons;>95% cells were positive (Supplementary Fig. 3). The isolated neurons were subjected to 2 h of OGD and then shifted to normal culture conditions. The cells were then divided into two groups, the NT group, in which cells were placed into a normal incubator (37 °C) for 24 h, or the MHT group, in which cells were placed in a 330 °C incubator for 12 h and then moved back to the normal incubator for another 12 h. Cell death in these groups was then analyzed by flow cytometry with propidium iodide. As shown in Fig. 1d, compared to the NT group, neuronal death in the MHT group cells was significantly suppressed after OGD/R for 24 h.

### MHT increased the expression of Sirt1 and LC3B but decreased p-P53 in cortical and cultured neurons after CA/CPR or OGD/R

**Fig. 2:**
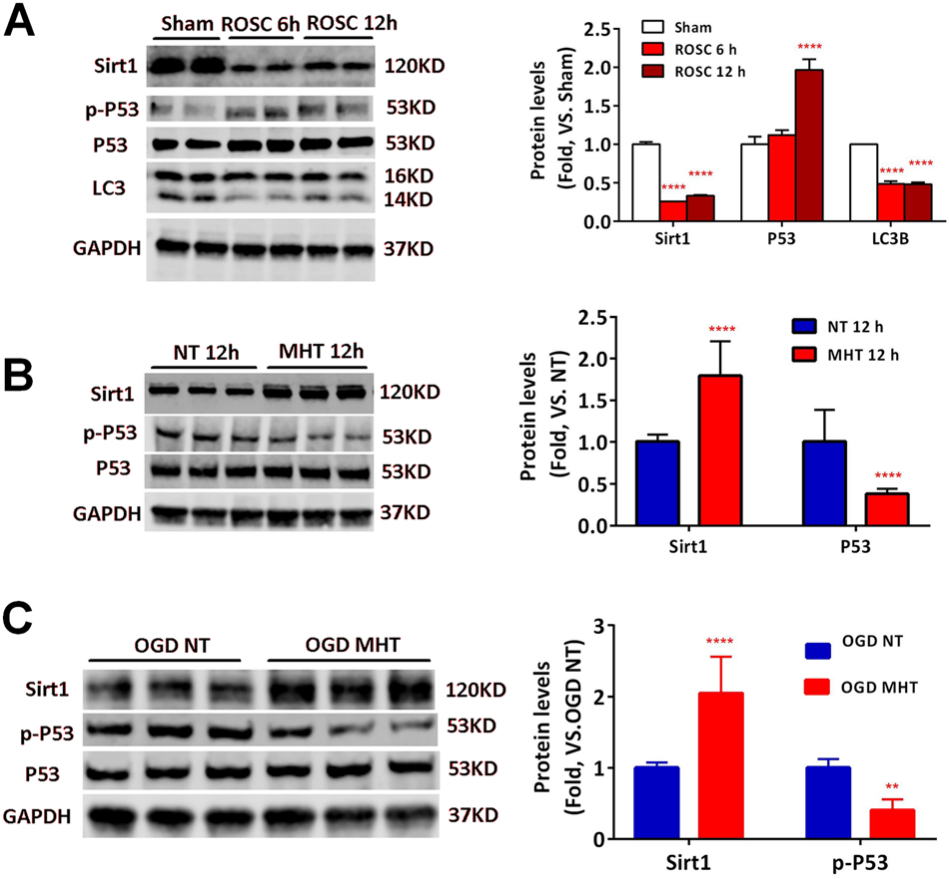
MHT increases expression of Sirt1 and LC3B and decreases p-P53 after CA/CPR and OGD/R both in vivo and in vitro. **a** Decreased expression of Sirt1 and LC3B but increased p-P53 in cortex of mice after ROSC for 6 h and 12 h. **b** MHT increases expression of Sirt1 and LC3B and decreases p-P53 in cortex of mice after ROSC for 12 h. **c** MHT increases expression of Sirt1 and LC3B and decreases p-P53 in cultured neurons after OGD/R for 12 h. *p < 0.05; **p < 0.01; ***p < 0.001; ****p < 0.0001, vs. NT (normothemia)

Previously, Sirt1 has been shown to exhibit neuroprotective properties in an array of neurological disorders, such as Alzheimer’s disease, Parkinson’s disease, and Huntington’s disease^20^. Mechanistically, Sirt1 inhibits neuronal apoptosis or deacetylates FoxO1 or P53 and promotes autophagy^13^. We examined the expression of Sirt1 in the cortex of mice from sham, ROSC 6 and 12 h mice, respectively, and found that Sirt1 expression was significantly suppressed by ROSC, which coincided with increased p-P53 and decreased LC3B expression (Fig. 2a). We also found that 12 h of MHT increased the Sirt1 and LC3B protein levels and decreased p-P53 in mouse cortex after ROSC compared with 12 h of NT (Fig. 2b). Similar results were found in cultured neurons after OGD/R for 12 h (Fig. 2c).

### MHT increased expression of autophagic gene expression and autophagic flux in cortex and cultured neurons after CA/CPR or OGD/R

**Fig. 3:**
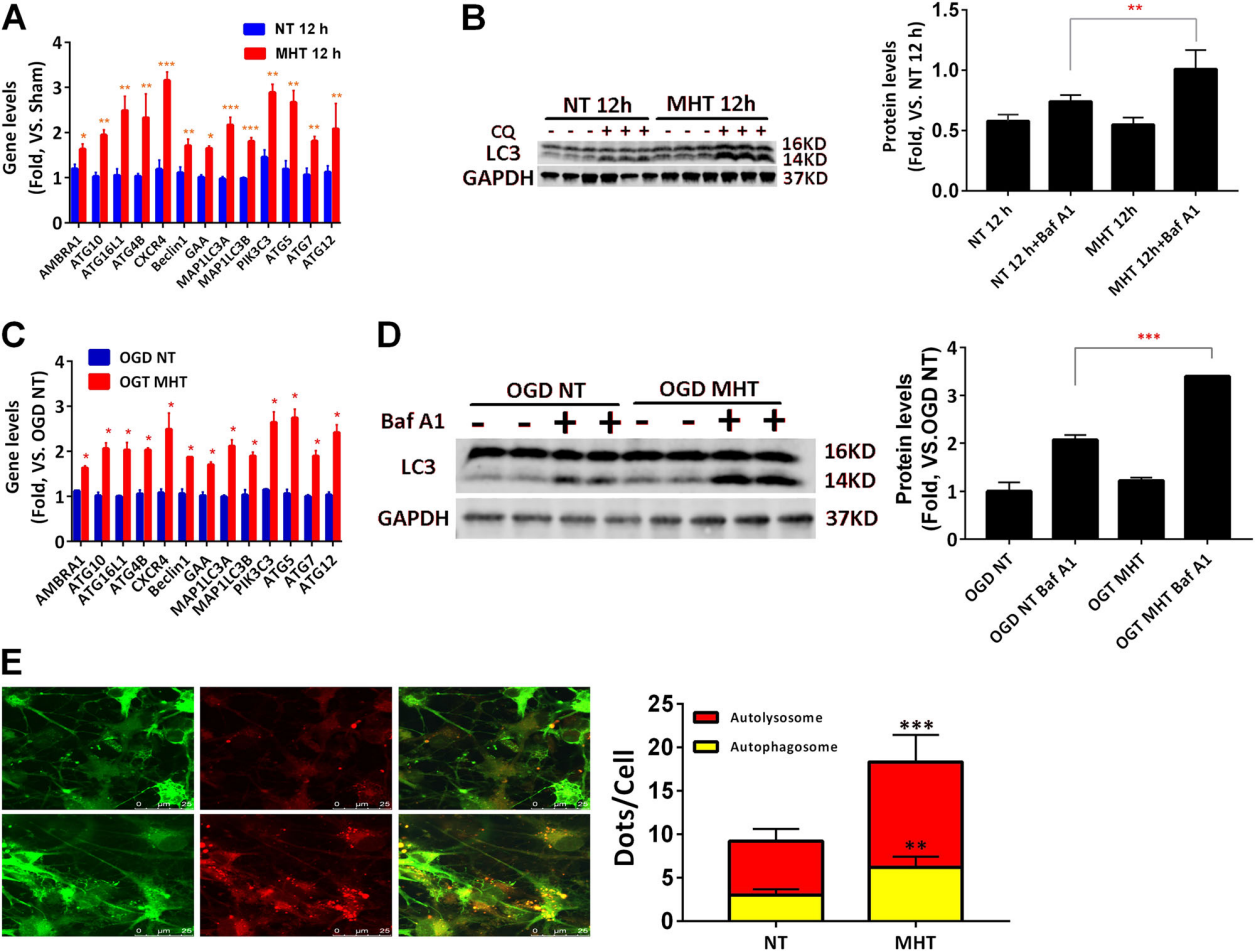
MHT increases autophagic flux in mice after CA/CPR and in neurons after OGD/R for 12 h. **a** MHT increases transcription of a subset of autophagic gene (AMBRA1, ATG10, ATG1L1, ATG4B, CXCR4, BECLIN1, GAA, MAPLC3A, MAPLC3B, PIK3C3, ATG5, ATG7, ATG12) in cortex of mice after ROSC for 12 h. **b** MHT increases autophagic flux in the cortex of mice after ROSC for 12 h. **c** MHT increases expression of a subset of autophagic genes (AMBRA1, ATG10, ATG1L1, ATG4B, CXCR4, BECLIN1, GAA, MAPLC3A, MAPLC3B, PIK3C3, ATG5, ATG7, ATG12) in cultured cortical neurons after OGD/R for 12 h. **d** MHT increases autophagic flux in cultured cortical neurons after OGD/R for 12 h. **e** MHT increases the number of red and yellow puncta in cultured neurons after OGD/R for 12 h. *p < 0.05; **p < 0.01; ***p < 0.001; ****p < 0.0001, vs. respective control groups as indicated

Sirt1 is an important regulator of autophagy^14,15^. Given that MHT increased the expression of Sir1 and LC3B in cortical and cultured neurons after ROSC or OGD/R, we next examined whether MHT could change the autophagic flux in cortex and cultured neurons insulted by ROSC or OGD/R. We first analyzed the mRNA levels of 50 genes reported to be involved in the autophagic pathway^21–23^. A subset of autophagic genes (AMBRA1, ATG10, ATG1L1, ATG4B, CXCR4, BECLIN1, GAA, MAPLC3A, MAPLC3B, PIK3C3, ATG5, ATG7, ATG12) were significantly upregulated in the MHT group compared those in the NT group (Fig. 3A,C). Since autophagy is a dynamic process, we used the autophagy inhibitor chloroquine in mice and bafilomycin A1 in neurons to further assess the autophagic levels in our model systems. We found that MHT increased the autophagic flux both in mice and in neurons in response to ROSC or OGD/R(Fig. 3b, d).

Next, we tracked the dynamic process of autophagic flux in real time with the double fluorescence labeled LC3 through adenovirus-mediated expression. This process fluoresced red and green (yellow) puncta when forming an autophagosome but only red puncta (as the GFP is quenched by the acidic environment of the lysosome) when forming an autolysosome. As shown in Fig. 3e, MHT significantly increased the number of red and yellow puncta in neurons after OGD/R for 12 h compared with NT. In aggregates, these findings suggest that MHT increased the Sirt1 expression, which was accompanied by the elevated autophagic flux in cortex in vivo when insulted with ROSC, and in cultured neurons in vitro when subjected to OGD/R.

### Suppression of Sirt1 abolished the neuroprotection of MHT both in vivo and in vitro

**Fig. 4:**
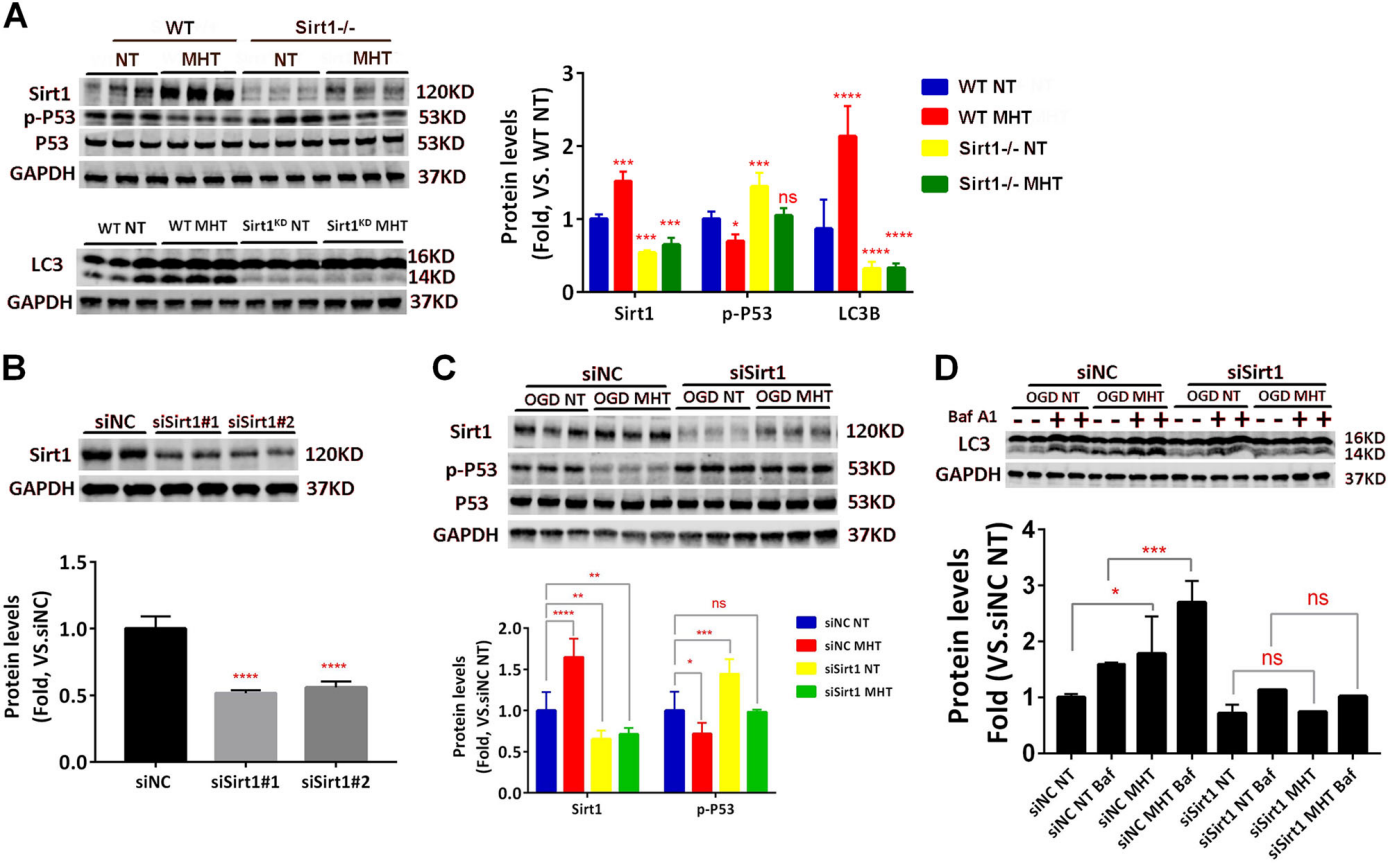
Suppression of Sirt1 reduces the MHT-induced increase in expression of LC3B and decrease in p-P53 level both in vivo and in vitro. **a** Sirt1 knockout attenuates the MHT-induced increase in LC3B expression and decrease in p-P53 level in cortex of mice. **b** Western blot showing Sirt1 knockdown efficiency by siSirt1 and siSirt2 in cultured neurons. GAPDH is used as a loading control. **c** Sirt1 knockdown increases p-P53 in cultured neurons. **d** Sirt1 knockdown blocks the autophagic flux in NT or MHT treated neurons after OGD/R. *p < 0.05; **p < 0.01; ***p < 0.001; ****p < 0.0001, vs. respective control group as indicated

To further establish a direct link between Sirt1 and MHT-induced neuronal protection during ROSC, we generated a CA/CPR model in Sirt1 knockout mice, which were then treated with either NT (Sirt1-/-:NT) or MHT (Sirt1-/-:MHT). While the wild type (WT):MHT group displayed significantly lower p-P53 compared to the WT:NT group, the Sirt1-/-:MHT group displayed significantly decreased LC3B protein levels and increased p-P53 levels in mice compared with the Sirt1-/-:NT group (Fig. 4a). Similar findings were obtained from the cultured neurons, which exhibited knockdown of the Sirt1 gene by siRNA, when subjected to OGD/R (Fig. 4b, c). Knockdown of Sirt1 also blocked the autophagic flux in neurons after OGD/R (Fig. 4d).

**Fig. 5:**
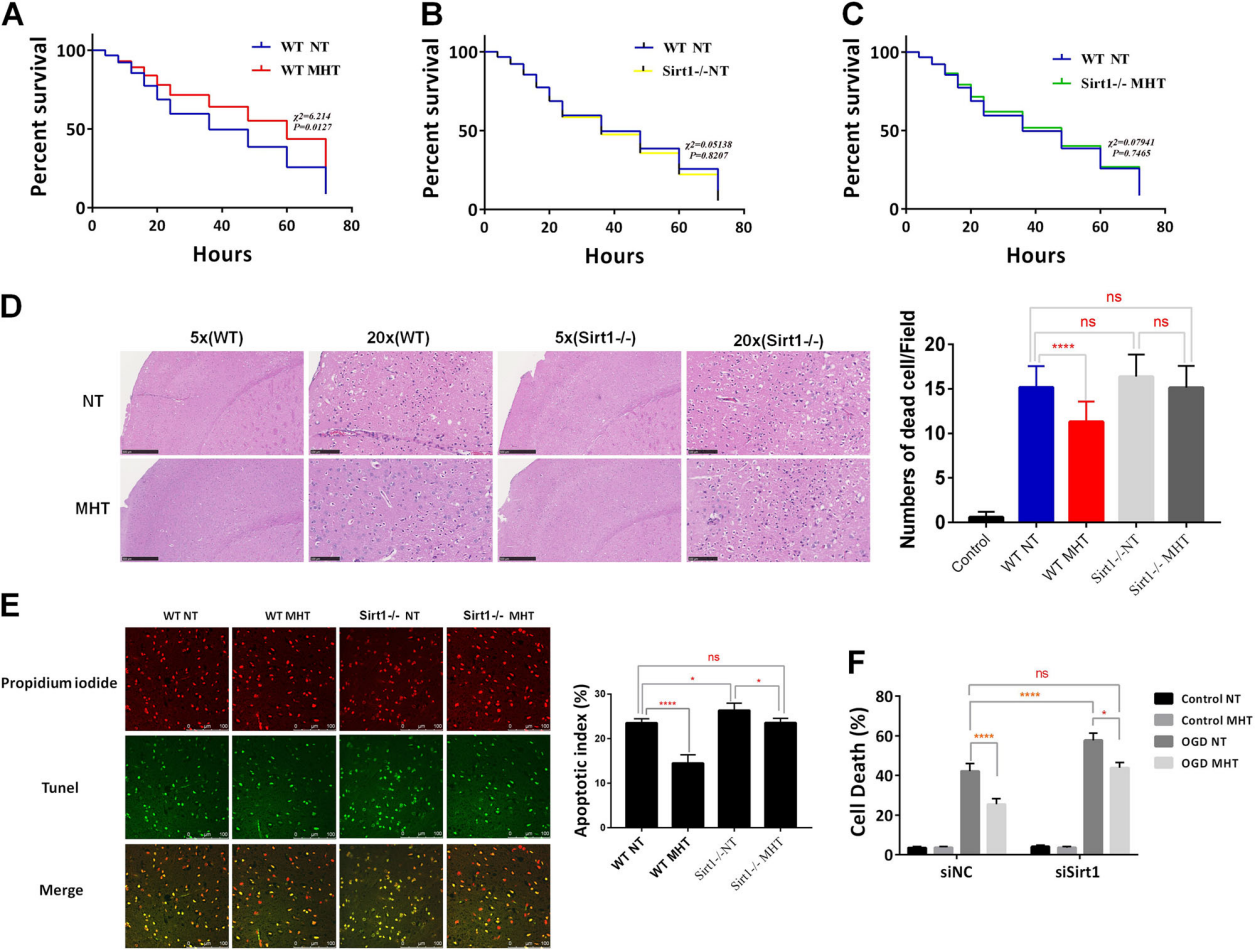
Suppression of Sirt1 abolishes the neuronal protection of MHT both in vivo and in vitro. **a** No difference in the survival rate between wild type and Sirt1 knockout mice after ROSC for 72 h. **b** Sirt1 knockout abolishes the MHT-improved survival rate of mice after ROSC for 72 h. **c** MHT does not significantly affect the survival rate of Sirt1 knockout mice. **d** Sirt1 knockout promotes cell death in the cortex of mice after ROSC for 72 h and abolishes the neuronal protection by MHT. **e** Sirt1 knockout increases the apoptosis in the cortex of mice after ROSC for 72 h and abolishes the inhibitory effects of MHT on neurons apoptosis (scale bar 100 µm). **f** Sirt1 knockdown promotes neuron death after OGD/R for 24 h and abolishes the neuronal protections by MHT. *p < 0.05; **p < 0.01; ***p < 0.001; ****p < 0.0001, vs. respective control groups as indicated

We next examined whether Sirt1 knockdown could affect the survival of mice subjected to ROSC. Sirt1 knockdown did not increase the death rate of mice after ROSC, but abolished the beneficial effects of MHT on the survival rate (Fig. 5a–c). Similarly, Sirt1 knockdown significantly increased apoptosis in NT and MHT neurons (Fig. 5d, e), as well as in the neurons in which OGD/R was applied (Fig. 5f).

### Increased autophagy contributes to MHT-induced neuroprotection

**Fig. 6:**
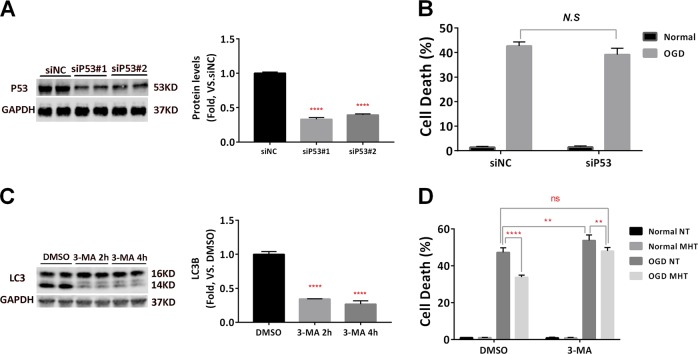
Suppression of autophagy attenuates MHT-reduced neuron death after OGD/R. **a** Western blot showing the efficiency in P53 knockdown by siRNAs in cultured neurons. **b** P53 knockdown has no significant effect on neuron survival after OGD/R for 24 h. **c** 3-MA inhibits autophagy in cultured neurons. **d** 3-MA treatment increases neuron death and abolishes neuroprotection by MHT after OGD/R for 24 h. *p < 0.05; **p < 0.01; ***p < 0.001; ****p < 0.0001, vs. respective control as indicated

To further elucidate the molecular basis leading to the neuronal protection of MHT, we knocked down P53 in cultured neurons (Fig. 6a) and subjected them to normal oxygenation or OGD/R. We found that the knockdown of P53 had no significant effect on neuron death after OGD/R (Fig. 6b), consistent with the recently published study^24^. We next used 3-MA, an important inhibitor of autophagy by means of blocking autophagosome formation^25,26^, to inhibit autophagy in neurons (Fig. 6c). The neurons treated with 3-MA suffered from increased apoptosis and abolished the neuronal protection by MHT after 24 h of OGD/R (Fig. 6d).

## Discussion

The present study investigated the mechanisms leading to the neuronal protection of MHT. The major findings from this study include that: (1) MHT significantly increases the survival rate of mice after CA/CPR following ROSC, and decreases apoptosis of cerebral cortical neurons, (2) MHT reduces primary cultured neuronal cell death after OGD/R, (3) Sirt1 expression in the cerebral cortex of mice is significantly downregulated after CA/CPR, and is accompanied by the decreased expression of autophagy-related protein LC3B and increased expression of p-P53 (MHT inhibited p-P53 and increased autophagic flux in both vivo and vitro), (4) suppression of Sirt1 by either genetic knockout in mice or siRNA-mediated knockdown in cultured neurons attenuated the neuronal protection by MHT, and (5) direct inhibition of autophagy compromised the MHT-linked neuroprotection. To the best of our knowledge, this is the first study to demonstrate that Sirt1-induced autophagy plays an important role in the neuroprotection by MHT after ROSC.

Sirt1 has important neuroprotective effects, and is also an important regulator of autophagy as has been previously demonstrated^14,15^. Sirt1−/− mice displayed larger infarct size following permanent middle cerebral artery occlusion (pMCAo) compared to their WT counterparts^27^. In contrast, Sirt1 transgenic mice showed less hippocampal damage following bilateral common carotid artery occlusion than WT mice^28^. A similar protection was seen in the Sirt1-Tg mice in a bilateral common carotid artery stenosis model of hypoperfusion injury^29^. Also, Sirt1 levels are modulated by ischemic injury as well. For example, Sirt1 was upregulated in the peri-infarct area for up to 7 days following pMCAo in mice^27^. On the other hand, in rats subjected to transient focal ischemia with various reperfusion periods, Sirt1 was downregulated for 6 h after reperfusion, compared with non-reperfused animals^30^. In the present study using a transient global ischemia/reperfusion model, the Sirt1 expression levels were decreased at 6 and 12 h after reperfusion. The discrepancy between these findings could be explained by the different species and ischemia/reperfusion models used. In the present study, we used a Sirt1 loss of function approach and found that suppression of Sirt1 compromised the neuroprotection by MHT both in vivo and in vitro, further supporting the notion that Sirt1 is critically involved in MHT-mediated neuroprotection against CA/CPR following ROSC.

The regulation of Sirt1 activity is complex and multifactorial. The basal level of Sirt1 is regulated by the transcription factor E2F1 (E2F1) that directly binds to the Sirt1 promoter at a cognate site^31^. Hence, factors such as calorie restriction and cellular stresses that increase the transcriptional activity of E2F1 may upregulate the Sirt1 levels^31^. FOXO belongs to another group of transcription factors that activates Sirt1. FOXO1 binds to several consensus sites on the Sirt1 promoter and enables its transcription^32^, while starvation in mammal cells activates FOXO3a and consequently augments Sirt1 expression^33^. In the present study, while we found that MHT upregulated Sirt1 expression in the cortex of mice after ROSC, we did not further investigate which signaling molecule(s) is involved in this mediation. However, this warrants further exploration.

Although P53 is known to regulate apoptosis and autophagy, the role of P53 in cerebral ischemia/reperfusion injury remains up for debate^34,35^, likely due to the different animal species used and/or ischemia/reperfusion models generated for the various studies. In the severe ischemic model, the high expression of P53 persists, thereby continuously activating its downstream genes to promote apoptosis. In the model of transient ischemia/reperfusion, the upregulated P53 expression does not persist and thus its role in promoting apoptosis is limited^36^. In the present study, the period of cerebral blood flow interruption lasted about 8 min, and was then reversed by CPR. Thus, in our model, the ischemic brain duration was shorter than the regular stroke model. In addition, the p-P53 only increased at 12 h after ROSC, which can explain why P53 did not play an important role in neuronal death after ROSC. In the early stage of cerebral ischemia/reperfusion, the damage of neuronal cells mainly manifests as necrotic death. In the late stage, it is mainly characterized by delayed neuronal apoptosis^37,38^. However, which method of cell death, necrosis or apoptosis, is more important in the prognosis of neurological function after ROSC remains to be answered. In the current study, we found that MHT reduced both necrosis and apoptosis, which we believe should be one of the major mechanisms underlying MHT-mediated neuronal protection.

Recent studies have shown that activation of autophagy is associated with the neuroprotection in brain ischemia or reperfusion model^39,40^. The protective role of autophagy during reperfusion may be attributable to mitophagy-related mitochondrial clearance and inhibition of downstream apoptosis^41^. Rapamycin, an autophagy enhancer, protects against focal brain ischemia in rats, indicating that autophagy may be neuroprotective^42^. Autophagy activation is an important mechanism for the MHT-mediated benefits to neuronal injury both in vivo^11^ and in vitro^12^. Consistent with the above findings, our previous study also suggested that activation of autophagy displays neurological protections in a rat CA model^10^. In line with these findings, in the current study, we found that 3-MA blocked the protective effect of MHT on neurons after OGD/R, further supporting the premise that activation of autophagy is an important mechanism by which MHT exerts its protection on neuronal cells after OGD/R.

In conclusion, in the present study, we employed loss and gain of function approaches to demonstrate both in vitro and in vivo that MTH provides neuroprotection after CA/CPR following ROSC at least in part through Sirt1-activated autophagic flux.

## Materials and methods

This project was approved by the Animal Investigation Committee of Sun Yat-sen University and the animal protocol conformed with National Institutes of Health Guidelines for ethical animal research.

### Animals

C57 male mice (6–8 weeks old, weight 20.0–30.0 g) were provided by the Animal Laboratory of Sun Yat-Sen University with animal permit number SCXK (Yue) 2015–0029 and certification No. 44008500011874. *Sirt1* knockout mice (6–8 weeks old, weight 20.0–30.0 g) were provided by the Hubei Provincial Laboratory Animal Public Service Center with certification No. 34018402012771.

### Antibodies

Mouse antibodies against β-tubulin (ab78078), MAP2 (ab5392), NeuN (ab128886), Sirt1 (ab104833), LC3A/B (ab128025), and p-P53 (hosphor S15) (ab1431) were purchased from Abcam. The rabbit anti-β-actin (20536-1-AP) antibody was purchased from Proteintech. The goat anti-rabbit IgG (sc-2004) secondary antibody was purchased from Santa Curz Biotechnology. T rabbit anti-mouse IgG (A9044) secondary antibody was purchased from Sigma. The goat anti-mouse IgG (DyLight™ 488 conjugated) (#4316) secondary antibody was purchased from Cell Signaling Technology.

### Generation of mouse CA model

Adult (6–8-week-old) male C57B/6 mice were randomly divided into two groups after ROSC, a normothermia (NT) group, in which the animal body temperature was maintained at 37 °C (acceptable range, 36.5−37.5 °C), and a mild hypothermia therapy (MHT) group, in which the animal body temperature was maintained at 33 °C (acceptable range, 32−34 °C). All animals were then subjected to CA/CPR.

Briefly, mice were fasted overnight but had access to water. Anesthesia was then induced with 3% isoflurane and maintained with 1.5–2% isoflurane in oxygen-enriched air using a nose cone. A temperature probe was inserted into the rectum to monitor body temperature. A PE-10 catheter was inserted into the right jugular vein for drug administration. Needle electrodes were placed subcutaneously in the chest for continuous electrocardiogram (EKG) monitoring. Mice were intubated endotracheally and connected to a ventilator (MiniVent Ventilator, Harvard Apparatus) with a respiratory rate of 150 breaths/min, a tidal volume of 0.2 ml, and an inspiratory–expiratory ratio of 1:1. CA was induced with an injection of 50 μl KCl (0.5 M) via the jugular catheter and confirmed by asystole on EKG. The cerebral blood flow was detected using laser Doppler flowmetry (Perimed, PeriScan Pim3), and declined over 80% of baseline during CA. During CA, the endotracheal tube was disconnected, and anesthesia was discontinued. Resuscitation began eight minutes after the induction of CA by a slow injection of 0.5–1.0 ml epinephrine solution (16 μg epinephrine/ml 0.9% saline), chest compressions, and ventilation with 100% oxygen at a respiratory rate of 150 breaths/min. Chest compressions were stopped as soon as spontaneous circulation was restored. Resuscitation was abandoned if spontaneous circulation was not restored within 20 min. Mice were extubated after an adequate respiratory rate was recovered and were provided with access to water and food. NT animals underwent the same procedures as those in MTH group except that they did not receive KCl or epinephrine injections or chest compressions. Rectal temperature of mice was maintained at 37 °C (NT group, acceptable range, 36.5−37.5 °C) or 33 °C (MHT group, acceptable range, 32 −34 °C) using a thermostatically regulated heating blanket or ice packs after CPR. The specific number of animals in each group has been written into supplementary materials.

### Genotyping of Sirt1 knockout mice

PCR was used for genotyping Sirt1 knockout mice as detailed in Supplementary data 4. The DNA was amplified and authenticated by agarose gel electrophoresis using 1.0% agarose (Biowest, BY-R0100) gel.

### Hematoxylin and eosin (H&E) staining

Mouse brain tissue was collected, immediately fixed in 4% formaldehyde, dehydrated and paraffin embedded. Five micrometer thick sections were generated from these fixed brain tissues, followed by standard H&E staining. Histopathological examinations were performed by a senior pathologist at the hospital. Cells were considered dead when any one of the following morphological criteria was met: (1) the cell lost the integrity of its plasma membrane, (2) the cell, including its nucleus, underwent complete fragmentation into discrete bodies (which are frequently referred to as ‘apoptotic bodies’), and (3) its corpse (or its fragments) was engulfed by an adjacent cell^43^. The dead neurons in each cortex section were counted per 400 × 400-pixel region under 40× magnification by light microscopy (OLYMPUS BX51; OLYMPUS IMAGING CORP, Japan).

### TUNEL staining

TUNEL staining was performed using the In-Situ Cell Death Detection kit (Roche) according to the manufacturer’s instructions. Propidium iodide staining was performed to visualize nuclei after TUNEL reaction. At least five randomly selected fields were scored, and the percentage of TUNEL-positive nuclei was quantified using the Image J software.

### Cell culture

Whole brains were collected from 1-day old neonatal sprague dawley (SD) rats. The cerebral cortex was isolated, cut into 0.5 mm^3^ pieces and digested in 0.25% Trypsin-EDTA (Gibco, 930004) for 30 min at 37 °C to isolate individual primary cortex neurons (PNs). PNs were then planted on poly-D-lysine (40 μg/ml, P6407, Sigma)-coated plates for 5 h in high glucose DMEM (Gibco, 11960085) supplemented with 5% fetal bovine serum (FBS, Gibco, 1581729) and 1% penicillin/streptomycin (Hyclone, SV30010). Next, the planting medium was replaced by neurobasal-A medium supplemented with 1% GlutaMAX (Invitrogen, 35050-06), 2% B-27 (Invitrogen, 17504-044) and 1% penicillin/streptomycin. Twelve hours later, the medium was replaced again with neuron culture medium containing 2 µmol cytarabine for 24 h (to inhibit glial cells growth) and was then replenished with fresh culture medium.

### Immunofluorescence

The PNs planted on the sheet glass were fixed with 4% paraformaldehyde for 20 min, washed with 0.1 M phosphate-buffered saline (PBS), permeablized in 0.3% Triton X-100 (MP Biomedicals, #194854) for 20 min and rinsed with PBS. Cells were blocked in 5% bovine serum albumin (BSA) at room temperature for 1 h, then incubated with anti-MAP2 and anti-NeuN antibodies at 4 °C overnight. This was followed by another incubation with DyLight 488 conjugated secondary antibody (H + L) (1:200) in wet-cassette for 1 h at 37 °C. After nuclear staining with DAPI, cells were visualized under a confocal fluorescent microscope (Leica, TCS SP5II).

### siRNA

siRNAs were purchased from Invitrogen and reconstituted as a stock solution of 40 mM. Five days after plating, neurons were transfected with siRNA negative control (siNC), and siRNAs against Sirt1 (siSirt1) and P53 (siP53) using RNAi Max transfectant according to the manufacturer’s recommendations. Following 48 h of siRNA transfection, neurons were switched to a serum-free medium.

### Generation of cellular OGD/R model

PNs were assigned randomly to the following groups, OGD-NT and OGD-MHT, which were further divided into siCN and siSirt1 groups with each having NT and MHT treatment. PNs were bathed in an appropriate amount of anoxic liquid (3.283 g deoxyglucose, 0.596 g KCl, 0.150 g MgSO4, 0.525 g NaHCO3, 4.766 g HEPES, 7.313 g NaCl, 0.163 g KH2PO4, 0.133 g CaCl2, and 0.560 g sodium lactate dissolved in 1 L of ddH_2_O, pH 6.6). Cell culture plates were arranged in a modular incubator chamber (95% N_2_ and 5% CO_2_) (Billups-rothenberg, CA92014). The ventilation pipe was closed 5 min after the oxygen detector (SMART SENSOR, AS8801) reachs a steady state of 0.3%. Hypoxia was induced for 2 h at 37 °C to establish the OGD mode. Then, the ischemic medium was replaced with the complete medium. Cells were arranged on the 37 °C (NT treatment) or 34 °C (MHT treatment) incubator to re-oxygenate for 12 or 24 h.

### Reverse transcription and quantitative polymerase chain reaction (RT-qPCR)

RNA was purified from the brain tissue or PNs using Trizol (Invitrogen, 15596026). one microgram of total RNA was reverse transcribed into cDNA using the PrimeScript^™^ RT reagent Kit with gDNA Eraser (Takara, RR047A). qPCR was performed in a Real-Time PCR System (Bio-Rad, CFX96 touch) using SYBR® Premix Ex Taq™ II (Takara, RR820A). In every RT-qPCR test, melting curves were detected at the end of the experiment to examine the primer specificity. Relative mRNA transcription was normalized to *Actb* and evaluated using the *F* = 2^^(-△△Ct)^ formula. Primers were provided by Invitrogen and their sequences are listed in Supplementary data.

### Western blot

Cells were lysed in RIPA (Beyotime, P0013B) containing 1% phenylmethanesulfonyl fluoride (PMSF, Beyotime, ST506), and 1% protease/phosphatase inhibitor (PI/PHI, Thermo Scientific, 78440). Protein concentrations were determined using a Pierce BCA Protein Assay Kit (Beyotime, P0009). Twenty microgram of total protein was resolved in 10% SDS-PAGE and transferred onto 0.2 μm polyvinylidene fluoride (PVDF) membranes (Millipore, ISEQ00010). The membranes were subsequently blocked with 5% BSA (Sigma, A7030) at room temperature for 1 h, and incubated with the primary antibodies of interest at 4 ˚C overnight, followed by another incubation with the appropriate secondary polyclonal antibodies (HRP-labeled goat anti-rabbit Ig G) for 1 h at room temperature. GAPDH was used as a loading control. The protein bands were visualized with a chemiluminescent imaging analysis system (General Electric, ImageQuant Las4000mini).

### Measurement of autophagic flux

Adenoviruses expressing GFP (Ad-GFP, control) and mRFP-GFP-LC3 (Ad-mRFP-GFP-LC3) were provided by Hanbio. PNs were seeded onto slides, and infected with Ad-GFP or Ad-mRFP-GFP-LC3 at a multiplicity of infection (MOI) of 0, 50, 100, 200 or 400, respectively for 8 h. In 72 h, cells were visualized under the automatic inverted fluorescent microscope. After OGD/R, autophagic flow of PNs was detected using a laser scanning confocal microscope (Leica, TCS SP5II).

### Statistical analysis

Data are represented as mean ± standard deviation (SD). An independent test and an one-way ANOVA, followed by a Bonferroni test for multiple comparisons, was used to compare continuous and discrete variables between groups, respectively. For animal studies, normality was assessed via the Shapiro–Wilk and Anderson–Darling statistics. Normally distributed data were analyzed with parametric tests, while non-normal data were analyzed with nonparametric tests (Mann–Whitney *U* test for two group comparisons and the Kruskal–Wallis test for ≥ 3 groups followed by the Dunn test to correct for multiple comparisons). Kaplan–Meier methods were used for univariate survival analyses, while the log-rank test was used to make comparisons between and/or among groups based on the treatment modality. A *p* < 0.05 was considered statistically significant. All statistical analyses were performed using the SPSS 13.0 software.

## Supplementary information

supplementary data
