# Supplementary material for: Mild hypothermia improves neurological outcome in mice after cardiopulmonary resuscitation through Silent Information Regulator 1-actviated autophagy
Source: Cell Death Discov. 2019 Aug 13;5:129. doi: 10.1038/s41420-019-0209-z (PMC6690976; doi:10.1038/s41420-019-0209-z)
Supplement: Supplementary file 1 — supplementary data [file 41420_2019_209_MOESM1_ESM.docx]

**Supplementary figure 1. Flow chart showing the protocol for the induction of CA, CPR and ROSC followed by experimental procedures.**

**
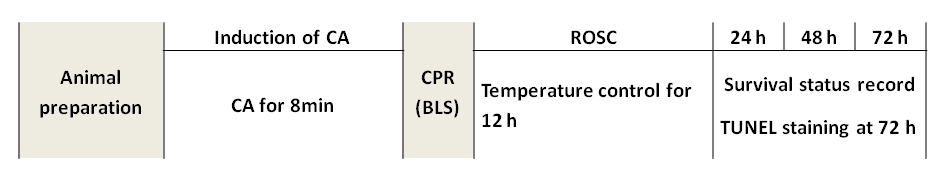
**

The first part mainly observes the effect of MHT on the neurological phenotype of mice (18 in each group) after ROSC, in this section we found that the mortality rate of animals after ROSC was extremely high, and the number of survivors to 72 hours was very small. The second part was to observe the expressions of Sirt1 after ROSC in another 18 mice, the results showed that the changes were most obvious after 12 hours after ROSC. In the third part, we used 18 mice in each group to observe the effect of MHT on the expression of Sirt1, p-P53 and LC3B (autophagy flux) at 12 hours after ROSC. The fourth part 18 Sirt1-/- mice in each group (NT and MHT) were used to observe the effects of Sirt1 knockdown on neurological phenotype, and then 18 Sirt1-/- mice in each group (NT and MHT) were used to observe P53 activation and autophagy activation in mice at 12 hours after ROSC.

**Supplementary figure 2. The basal physiological parameters of mice did not show any differences between NT group and MHT group.**

**Supplementary figure 3. Identification of neurons. Immunostaining against neuron markers, Neun and Map2, was performed as described in Materials and methods. DAPI, nuclear staining.**


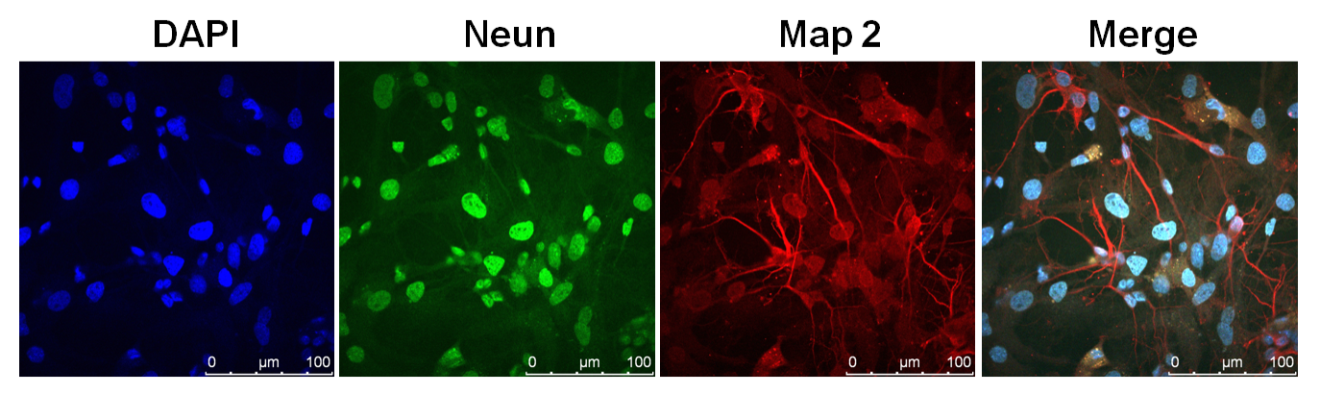


**Supplementary data 4. Protocol for genotyping Sirt knockout mice**

Strain name: Sirt1-flox

Primer seq:

Forward: GGT TGA CTT AGG TCT TGT CTG

Reverse: CGT CCC TTG TAA TGT TTC CC

| Step | Temp (℃) | Time (s) | Notes |
| --- | --- | --- | --- |
| 1 | 94 | 180 |  |
| 2 | 94 | 30 | Repeat for 35 cycles |
| 3 | 56 | 60 |  |
| 4 | 72 | 60 |  |
| 5 | 72 | 180 |  |

| Primer set | | Product size (bp) | Product |
| --- | --- | --- | --- |
| Primer1 | Primer 2 | 550 | WT |
| Primer1 | Primer 2 | 750 | floxed |

Marker f/f f/+ +/+ Marker


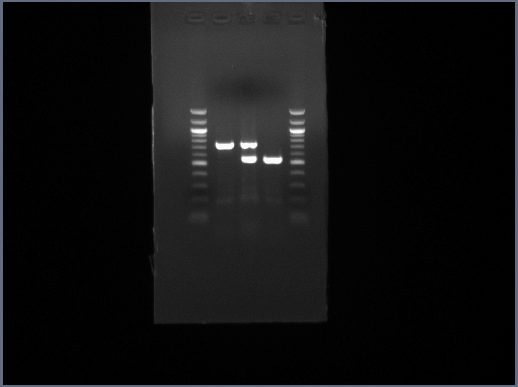


floxed: 750 bpWT: 550 bp

**Supplementary figure 5. The basal physiological parameters of mice did not display any significant differences among the four groups. (BLS=Base life support; CBF=Cerebral blood flow)**


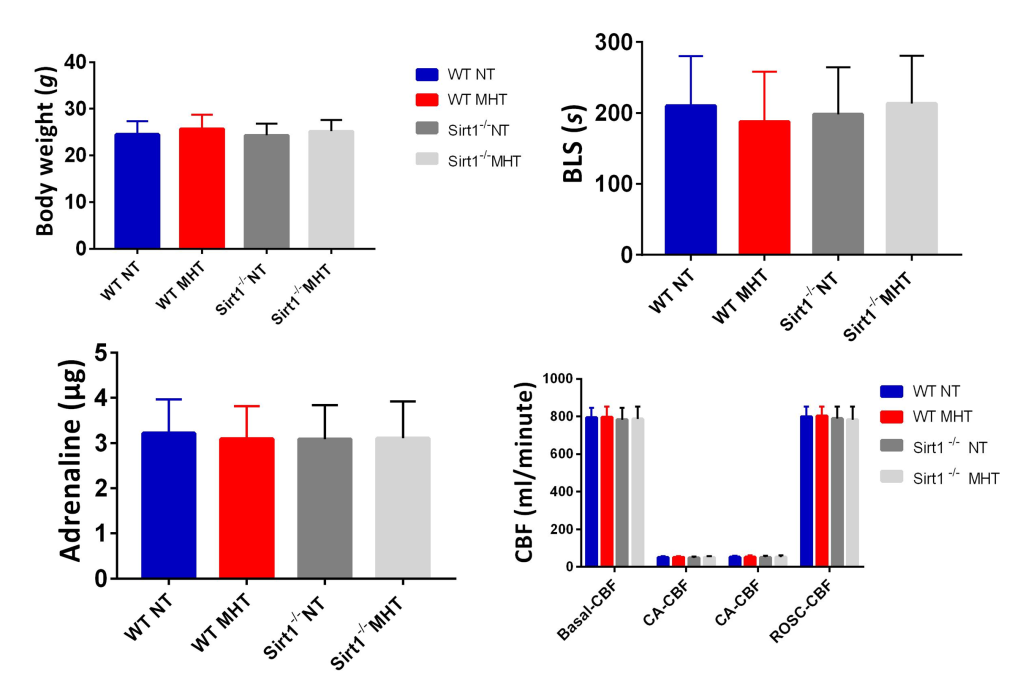


| RAT |  | AUTOPHAGY GENE |
| --- | --- | --- |
| AMBRA1 | F | GAGCCACGGAACCGATAGAG |
| AMBRA1 | R | CAGTTGGACTGCCCCTTACC |
| APP | F | GCATCCTTTTGGGGTGGACT |
| APP | R | ATGTCCGAACTCCGCATCC |
| ARSA | F | CTTCCCACCACACCCACTAC |
| ARSA | R | ACGCATCAATTCAGGACCGT |
| **ATG10** | **F** | CTCCAACACCACATCGGGAA |
| **ATG10** | **R** | ACAGAACGGGCACTTGGTAG |
| ATG12 | F | ATTGACACACTGGCGGACTT |
| ATG12 | R | AATCCCCTTGGCTCACGTTT |
| ATG16L1 | F | TCTATCAATGCGGTGGCGTG |
| ATG16L1 | R | AAGGTACGGTGGGTGCCATA |
| ATG16L2 | F | ATGCTGTTTGTTTTGCCCCC |
| ATG16L2 | R | AGCCTGGTTGTAAGTAGCCG |
| ATG3 | F | TGGAGCTTGGGGGACGAAA |
| ATG3 | R | TGGCATCGAATCCCGCA |
| ATG4A | **F** | TGTGTTCTTCCTGTGGGTGC |
| ATG4A | **R** | CAATGAGCAGCAGGGGTTTC |
| ATG4B | F | AGACAGAGCAGGTAGCTCCA |
| ATG4B | R | CCCAGGAAGCCAAGGTTAGG |
| ATG4C | F | CGAAAAGAGCAACGAGCGAG |
| ATG4C | R | AGCAAGAGCCATCAAGTCGT |
| ATG4D | F | TGCCCTTTCGAGTCCTCAC |
| ATG4D | R | CAGTCAGTCAGTCCTCCCAGT |
| ATG9A | F | CTTACCTCCAGTCCTCACCC |
| ATG9A | R | AAAGGTAGTGTGAGGGGAGC |
| ATG9B | **F** | AGGACCACCAACCTTTGCTC |
| ATG9B | **R** | AACCGTTCCGCTGGTGATAG |
| BAD | F | TCCTGGGGAGCATCGTTCAG |
| BAD | R | GGTACGAACTGTGGCGACT |
| BAK | F | TCCTGGGGAGCATCGTTCAG |
| BAK | R | GGTACGAACTGTGGCGACT |
| BAX | F | TTTGTGGCTGGAGTCCTCAC |
| BAX | R | TTCCCCGTTCCCCATTCATC |
| BCL2 | F | CTCATGCCAAGGGGGAAACA |
| BCL3 | R | ACCAATAGCACTTCGCGTCC |
| BID | **F** | CACCATCTCTGCTCCGTGAC |
| BID | **R** | AGTCACAGTTCCGTTGGAGG |
| BNIP3 | F | AGCATGAGAAACACAAGCGT |
| BNIP3 | R | TGAGCAGAAGGCAGATCCAAG |
| CLN3 | F | TACCAGCTGGGACCTCTCTC |
| CLN3 | R | GCTCAAAAAGCCCCTGGTTG |
| CXCR4 | F | GGAAGGAACTGAACGCTCCA |
| CXCR4 | R | ACAATTCAGGAACGGGGTGG |
| DRAM | F | TGTGAGTGGACCGTTGCTTT |
| DRAM | R | CGTCCGTCCTCTGTCACTTC |
| EIF2AK3-F | F | TACAAGGCTGTCACTCAGGTG |
| EIF2AK3-R | R | CCTTGGAGCACCGAAAGCG |
| EIF4G1-F | F | CCCTCTTTCAGCTTGTGCTTC |
| EIF4G1-R | R | AGGTGGAGTGGGAGAGACAAT |
| FAM176A-F | F | GGGGGAGACCGAAGGAAATG |
| FAM176A-R | R | TCAAGACTGAGCAAGGGACG |
| GAA -F | F | ACCAAGGAGGGAGGTGAACT |
| GAA-R | R | CAGGGATGGACAGGCTCTTG |
| GABARAP-F | F | AAAGCTCGGATAGGGGACCT |
| GABARAP-R | R | CACTGGTGGGTGGAATGACA |
| GABARAPL1-F | F | GTGTCTACGGGAAATGAGGCA |
| GABARAPL1-R | R | TGTGCGGTGTGATGTTTCCT |
| GABARAPL2-F | F | AATGAAGGGCGGTAGGTGTG |
| GABARAPL2 -R | R | GCCGTCTAACACTCCGAACA |
| HGS-F | F | CCATCGGTGCAGAGTACAGT |
| HGS -R | R | TCATAGCAGGGCTCACACAC |
| HTT -F | F | AGGTGGTGGTCTCAATGCTG |
| HTT -R | R | CTTGGCTAACATGGGCAGGA |
| MAP1LC3A -F | F | CTCCCAAGAAACCTTCGGCT |
| MAP1LC3A-R | R | GACTTGGTATGCTGGCTGGT |
| MAP1LC3B-F | F | TCCTGAACCCCAGCCATTTC |
| MAP1LC3B -R | R | GGCATGGACCAGAGAAGTCC |
| PIK3C3 -F | F | ATCCCATTGCCGTTAGAGCC |
| PIK3C3 -R | R | GCCACCGTCTTCTGTCTTGA |
| PIK3R4 -F | F | AGAGATGGGATTGTGAAGGTGT |
| PIK3R4 -R | R | CCCACATCCTTACCCACTGT |
| PTEN -F | F | AGGAGTATCTTGTGCTCACCC |
| PTEN -R | R | TGGAGAGAAGTATCGGTTGGC |
| RAB24 -F | F | ACAGGATCGGCCACTTCCG |
| RAB24-R | R | GCTAACTCCAAACAGCACCTC |
| RGS19 -F | F | GGACCTCCCAGTCGAAATCC |
| RGS19 -R | R | TGCCCAGCTCTGTACTTCCT |
| SNCA -F | F | GGGAGTCGTTCATGGAGTGA |
| SNCA -R | R | GTACCCTTCTTCACCCTTGCC |
| TP53 -F | F | TGAGGTTCGTGTTTGTGCCT |
| TP53 -R | R | TCCGGGCAATGCTCTTCTTT |
| ULK1 -F | F | CCCTCGTTTGACTTCCCCAA |
| ULK1 -R | R | GGTTCCGAGGTGGTGTCATT |
| UVRAG -F | F | CGGACTGATGGTCAGATGGT |
| UVRAG -R | R | CAGGTACAGAGCAATGCGGG |
| VPS11 -F | F | CGAGGGGTTTTCAACAAGCTG |
| VPS11 -R | R | GTCTGCTACCGGATGTCCCT |
| VPS18 -F | F | TCATTCCTGCCCTGGTGAAC |
| VPS18 -R | R | AGTCTCGCCAAGCACATTCA |
| SQSTM1 -F | F | GATAGCCTTGGAGTCGGTGG |
| SQSTM1 -R | R | AGACAAATGCGTCCAGTCGT |
| ATG5 | F | GTGATCCCGGTAGACCCAAC |
| ATG5 | R | AAACCACACGTCTCGAAGCA |
| ATG6 | F | CTCGTCAAGGCGTCACTTCT |
| ATG6 | R | GCCTTAGACCCCTCCATTCC |
| ATG7 | F | GAGACCGTCTGAGCAACCAC |
| ATG7 | R | ATGACACAGGAAAGGGTGCAA |
| 18s | F | TCTCAAGTGGTCCACAAGGC |
| 18s | R | TGGGCTTAACTGGGGTTAGT |

| mice |  |
| --- | --- |
| ATG10.F | AGCTGCCAGTGTGCTTACAT |
| ATG10.R | TCCTCCAGAGCTAACGGTCT |
| ATG12.F | TAAACTGGTGGCCTCGGAAC |
| ATG12.R | ATCCCCATGCCTGGGATTTG |
| ATG3.F | ACCACTGTCCAACATGGCAA |
| ATG3.R | TACCCATCCCCCATCACCAT |
| ATG4B.F | AGATGGACGCAGCCACTTT |
| ATG4B.R | CCAGTTCCCCCAATAGCTGG |
| ATG4C.F | TGCGCGGCTGTACAGTTATC |
| ATG4C.R | AATCCAATATACTTCTCCACTCCG |
| ATG9A.F | TGTCTCTTGTCCACCCTATCCT |
| ATG9A.R | GGCCACCATGTAGTTCTGGA |
| BNIP3 .F | GCCATTGGCTTGGGGATCTA |
| BNIP3 .R | TGCAAACACCCAAGGACCAT |
| EIF2AK3.F | CACGCAGATCACAGTCAGGT |
| EIF2AK3.R | GTGGGGCTGAGGATGGAAAA |
| MAP1LC3A .F | TACATGGTCTACGCCTCCCA |
| MAP1LC3A .R | GCCTAATCCACTGGGGACTG |
| VPS11.F | TGGGGCTCCTTATGCTGTTT |
| VPS11 .R | TGAGCCAACCCATCACTGTC |
| VPS18 .F | CACACGGGAGTGTTTCCGTA |
| VPS18 .R | ATGTGCTCTGTGTCTCCGTG |
